# The BDNF Val66Met Polymorphism Affects the Vulnerability of the Brain Structural Network

**DOI:** 10.3389/fnhum.2017.00400

**Published:** 2017-08-03

**Authors:** Chang-hyun Park, Jungyoon Kim, Eun Namgung, Do-Wan Lee, Geon Ha Kim, Myeongju Kim, Nayeon Kim, Tammy D. Kim, Seunghee Kim, In Kyoon Lyoo, Sujung Yoon

**Affiliations:** ^1^Department of Psychiatry, Catholic University of Korea College of Medicine Seoul, South Korea; ^2^Ewha Brain Institute, Ewha Womans University Seoul, South Korea; ^3^Department of Brain and Cognitive Sciences, Ewha Womans University Seoul, South Korea; ^4^Department of Neurology, School of Medicine, Ewha Womans University Seoul, South Korea; ^5^Graduate School of Pharmaceutical Sciences, Ewha Womans University Seoul, South Korea

**Keywords:** BDNF Val66Met, network resilience, white matter structural network, diffusion tensor imaging, tractography

## Abstract

Val66Met, a naturally occurring polymorphism in the human brain-derived neurotrophic factor (BDNF) gene resulting in a valine (Val) to methionine (Met) substitution at codon 66, plays an important role in neuroplasticity. While the effect of the BDNF Val66Met polymorphism on local brain structures has previously been examined, its impact on the configuration of the graph-based white matter structural networks is yet to be investigated. In the current study, we assessed the effect of the BDNF polymorphism on the network properties and robustness of the graph-based white matter structural networks. Graph theory was employed to investigate the structural connectivity derived from white matter tractography in two groups, Val homozygotes (*n* = 18) and Met-allele carriers (*n* = 55). Although there were no differences in the global network measures including global efficiency, local efficiency, and modularity between the two genotype groups, we found the effect of the BDNF Val66Met polymorphism on the robustness properties of the white matter structural networks. Specifically, the white matter structural networks of the Met-allele carrier group showed higher vulnerability to targeted removal of central nodes as compared with those of the Val homozygote group. These findings suggest that the central role of the BDNF Val66Met polymorphism in regards to neuroplasticity may be associated with inherent differences in the robustness of the white matter structural network according to the genetic variants. Furthermore, greater susceptibility to brain disorders in Met-allele carriers may be understood as being due to their limited stability in white matter structural connectivity.

## Introduction

The brain-derived neurotrophic factor (BDNF) protein is a member of the nerve growth factor family of neurotrophins and is known to be essential for the development and maintenance of the neurons (Binder and Scharfman, 2004). A common single nucleotide polymorphism of rs6265 in the BDNF gene causes a substitution of valine (Val) to methionine (Met) at codon 66 in the prodomain (Val66Met), which influences activity-dependent release of the BDNF protein (Kuczewski et al., 2010). The BDNF Val66Met polymorphism has been reported to be associated with cognitive (Egan et al., 2003; Hariri et al., 2003) and emotional (Chen et al., 2006; Soliman et al., 2010) dysfunctions by modifying cerebral cortex excitability (Kleim et al., 2006), gray matter structures (Frodl et al., 2007; Harrisberger et al., 2015), or white matter integrities (Pezawas et al., 2004; Ho et al., 2006). Furthermore, a previous study on a rodent model indicated that the BDNF Val66Met polymorphism is associated with the modulation of glutamate receptor activities, which then undergoes alterations in the hippocampal long-term depression (Mizui et al., 2015).

More specifically, the relationships of the BDNF Val66Met polymorphism with cognition (Egan et al., 2003; Hariri et al., 2003; Pezawas et al., 2004; Ho et al., 2006; Montag et al., 2009), emotion (Chen et al., 2006) and even with several brain disorders including major depression (Dalby et al., 2013; Choi et al., 2015), epilepsy (Chen et al., 2016), schizophrenia (Ho et al., 2006), and stroke (Ramos-Cejudo et al., 2015) are suggested to be mediated by its effects on the alterations in gray and white matters. For instance, Met-allele carriers, in comparison to Val homozygotes, had gray matter volume deficits found in the temporal, frontal areas and thalamus (Pezawas et al., 2004; Ho et al., 2006; Montag et al., 2009). Val homozygotes on the other hand, have shown lower white matter integrities of fiber tracts in the frontal, temporal, and occipital areas in comparison with Met-allele carriers (Chiang et al., 2011; Tost et al., 2013).

Despite having a relatively large amount of evidence supporting the relationship between the BDNF Val66Met polymorphism and gray/white matter structural alterations, its effects on the graph theory-based white matter connectivity are largely unknown. Given that nodes (as brain regions) and edges (as physical connections between brain regions) are key components in the graph theory-based network analysis (Sporns, 2012), it would be necessary to investigate the white matter network endophenotype of the BDNF Val66Met polymorphism. Furthermore, when considering the brain as a network that displays similar behavior to other complex systems (Bullmore and Sporns, 2009), white matter network topology is suggested to organize a variety of brain functions and to be related to several brain disorders (Sporns, 2012). In this respect, the pivotal role of the BDNF Val66Met polymorphism in the human brain could be understood in terms of the impact of the genetic factor on the white matter network configuration of the brain.

In this study, we sought to examine the impact of the BDNF Val66Met polymorphism on the topologic configurations of the graph-based white matter structural network using deterministic tractography. In particular, we have focused on the network robustness that addresses organizational stability of a complex network system (Albert et al., 2000) by assessing the error and attack tolerance of the white matter structural networks against random failures and targeted attacks, respectively. This process was done by simulating damages to gray matter regions (nodes) or white matter connections (edges). We hypothesized that the central role of the BDNF Val66Met polymorphism in brain functions could be linked to genetically inherent differences in the white matter structural network configuration according to the genotypes of the BDNF gene.

## Materials and Methods

### Participants

Healthy individuals who had no lifetime history of substance abuse other than nicotine abuse, and were free of any psychiatric disorders that meet the Structured Clinical Interview for DSM-IV Axis I Disorders criteria were enrolled in the study. In addition, presence of any clinically significant medical diseases or any contraindications to magnetic resonance imaging were also an exclusion criteria for study participation. Demographic characteristics of the study participants are presented in Table 1. Among 74 healthy controls who were initially recruited, 73 participants who had T1- and diffusion-weighted images with the quality adequate for further analyses were included in the study. All participants voluntarily provided written informed consent in accordance with the Declaration of Helsinki and its later amendments. The study protocol was approved by the institutional review board of the Catholic University of Korea College of Medicine.

**Table 1:** Characteristics of study participants.

	Val homozygotes (*n* = 18)	Met-allele carriers (*n* = 55)
*Demographic characteristics*		
Age—year	43.6 ± 8.3	41.1 ± 12.7
Female sex—no. (%)	12 (66.7)	31 (56.4)
Race/Ethnicity—no. (%)		
East Asian	18 (100)	55 (100)
Right handedness—no. (%)	16 (88.9)	47 (85.5)
*Smoking*		
Current smoker—no. (%)	2 (11.1)	8 (14.6)
*Network characteristics*		
Global efficiency	0.556 ± 0.007	0.552 ± 0.015
Local efficiency	0.748 ± 0.011	0.750 ± 0.013
Modularity	0.329 ± 0.019	0.343 ± 0.035

*Means and standard deviation values are denoted as mean ± standard deviation*.

### Genotyping

Genomic DNA was extracted using the Promega genomic DNA purification kit and extracted DNA was quantified on a spectrofluorometer (Victor 2, Perkin Elmer, Waltham, MA, USA) using the PicoGreen dsDNA quantification kit. For the BDNF Val66Met polymorphism, DNA was genotyped using the polymerase chain reaction procedure. Based on the genotyping of the variant of the BDNF gene, 13 participants were identified as carriers of the Met/Met genotype, 42 participants as carriers of the Val/Met genotype, and 18 participants as carriers of the Val/Val genotype. The BDNF Val66Met polymorphism is known to have its allele frequencies dependent on ethnicity (Yeebo, 2015), and the allele frequencies for the Asian (Korean) participants in this study broadly agreed with those in Asian populations (Miura et al., 2014). Due to the relatively small number of Val/Met genotype carriers as analogous to previous imaging genetic studies (Tost et al., 2013; Kim et al., 2016), we merged Val/Met and Met/Met genotype carriers into a single genotype group to consider two genotype groups for subsequent analyses: the Met-allele carrier group (*n* = 55) and the Val homozygote group (*n* = 18).

### Acquisition of Imaging Data

Diffusion tensor imaging (DTI) data were collected using a Signa HDx 1.5T magnetic resonance imaging system (GE Healthcare, Milwaukee, WI, USA). In each participant, 60 images were acquired with a diffusion-weighted echo planar imaging sequence: echo time = 84 ms, repetition time = 17,000 ms, flip angle = 90°, and number of excitation = 2. The data set consisted of 54 images with high diffusion weighting (*b* = 1000 s/mm^2^) and six images with no diffusion weighting. Each image included 68 axial slices of 2.30 mm thickness with 96 × 96 matrix size and 2.29 × 2.29 mm in-plane resolution. Inspection of gross structural abnormalities and rating for image quality were performed by a neuroradiologist, who was blind to each individual’s clinical information. Among 74 T1- and diffusion-weighted images, one image was excluded from further analyses due to motion artifact which deemed the image as inadequate in quality.

### White Matter Tractography

Images were realigned to the non-diffusion-weighted image to correct for eddy-current-induced distortions and head motion. Diffusion tensor fitting and fiber tracking of DTI data were conducted using the Diffusion Toolkit^1^. White matter tracts were reconstructed over the whole brain using a deterministic streamline approach based on the Fiber Assignment by Continuous Tracking (FACT) algorithm (Mori et al., 1999). A diffusion tensor was modeled and the principal diffusion direction was estimated at each voxel. Each streamline from the seed followed the main diffusion direction and was terminated if a fiber tract had voxel-wise values of fractional anisotropy at threshold of 0.1, or the streamline turned at an angle >45°. Streamlines shorter than 10 mm were removed from further analyses, as these were regarded as being spurious.

### Construction of the White Matter Structural Network

In order to determine the cortical and subcortical nodes for constructing the white matter structural networks, T1-weighted images were preprocessed and parcellated into 82 different regions (34 cortical and seven subcortical regions per each hemisphere) using the FreeSurfer tool^2^ (Desikan et al., 2006). The averaged non-diffusion image (*b* = 0 s/m^2^) of each subject was linearly registered to the corresponding T1-weighted image. A set of 82 cortical and subcortical masks were then inversely transformed to the DTI native space.

An 82 × 82 connectivity matrix consisting of a set of nodes which reflect cortical and subcortical parcellated regions, as well as edges which reflect reconstructed connections between nodes was obtained from each individual. A minimum of three streamlines interconnecting two different nodes were required in order to be considered as being structurally connected. The connectivity matrix was converted to an adjacency matrix in a binary fashion that consisted of 1’s and 0’s, where its value was determined by whether fiber tracts between pairs of nodes existed (tract number ≥3) or not (tract number <3). This adjacency matrix represented an unweighted and undirected network based on white matter structural connectivity in such a way that 1’s corresponded to edges and 0’s to anti-edges. Participant-wise network construction following this procedure yielded 55 and 18 white matter structural networks for the MET and VAL groups, respectively.

### Assessment of Global Network Measures

Using the Brain Connectivity Toolbox^3^, the global topological organization of each whole-brain white matter structural connectivity network was examined. Specifically, the global efficiency and local efficiency were computed in each matrix. Global efficiency (*E_glob_*) is defined as the average of the inverse of the shortest length paths between all pairs of nodes, and local efficiency (*E*_loc_) is a measure of segregation of structural connectivity network. Global efficiency reflects the capacity for communication at the network level, whereas local efficiency calculates the efficiency on node neighborhoods. In addition, modularity which reflects the degree of the strength of division of a network into the non-overlapping groups of nodes, was also investigated (Rubinov and Sporns, 2010).

### Assessment of Network Robustness Measures

Network robustness of each individual matrix was assessed in response to continuous damage to nodes or edges. For the white matter structural network as constructed in the current study, damage to nodes and edges can be understood as lesions in the gray matter regions and interregional white matter connections, respectively. Two different kinds of damage or lesions were considered in order to assess the error and attack tolerance of the white matter structural network: random failures and targeted attacks. Random failures were simulated by randomly selecting and continuously removing nodes (Albert et al., 2000) or edges (Kaiser and Hilgetag, 2004) from the undamaged network. This process of removing nodes or edges was repeated 1000 times with randomly selected elimination orders. In contrast, targeted attacks were simulated by continuously eliminating specific nodes or edges which were chosen according to the betweenness centrality of nodes and edges in decreasing order. The measures of robustness were selected from referring to previous studies on network robustness, and consisted of the largest component size (Achard et al., 2006; He et al., 2008) and global efficiency (Achard et al., 2006; van den Heuvel and Sporns, 2011). In the current study, changes in robustness are visualized using line graphs, where the largest component size or global efficiency is a function of the number of nodes or edges removed. Network robustness measures in each individual graph are calculated as areas under the curve (AUCs) of the largest component size and global efficiency. More robustness in white matter structural networks indicate a larger connected component or a greater global efficiency even when several nodes or edges are removed. Therefore, a larger AUC for each individual graph represents a greater robustness of white matter structural networks.

### Statistical Analysis

Demographic characteristics were compared using the two-paired *t*-test, chi-square test and Fisher exact test.

For the assessment of group differences in global network measures (*E*_glob_, *E*_loc_ and modularity), multiple linear regression analysis was used after adjusting for age. A Bonferroni correction was applied to correct for multiple comparisons of group differences in the global network measures (*p* < 0.05/3).

Group differences in network robustness measures (AUCs of the largest component size and global efficiency at node and edge attacks) were examined using the multiple regression analysis after adjusting for age. The level of significance was set as a Bonferroni corrected *p* value of 0.05/4 for both random and targeted attacks.

Effect size for between-group differences in global network measures and network robustness measures was calculated using Cohen’s* d*.

## Results

There were no differences in age (*t* = 0.79, *p* = 0.43), sex composition (*χ*^2^ = 0.59, *p* = 0.44), handedness (Fisher exact probability test, *p* = 1.0) and smoking status (Fisher exact probability test, *p* = 0.53) between Val homozygotes and Met-allele carriers (Table 1). All participants were recruited in South Korea and were of East Asian ethnicity.

### Between-Group Differences in Global Network Measures

Table 1 summarizes the between-group differences in global network measures including *E_glob_*, *E*_loc_ and modularity. There were no differences in *E*_glob_ (*β* = 0.17, Bonferroni-corrected *p* > 0.1, effect size = 0.35), *E*_loc_ (*β* = −0.05, Bonferroni-corrected *p* > 0.1, effect size = 0.14), or modularity (*β* = −0.20, Bonferroni-corrected *p* > 0.1, effect size = 0.43) between Val homozygotes and Met-allele carriers.

**Figure 1:**
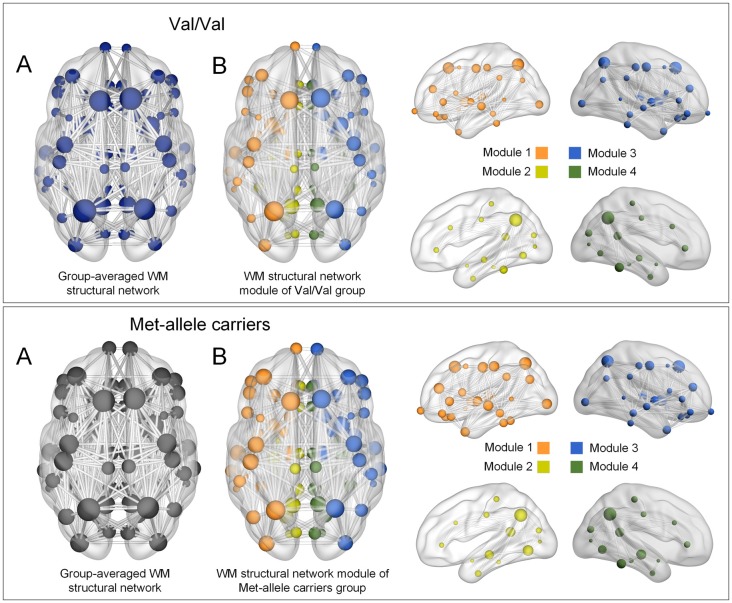
**(A)** Group-averaged reconstructed white matter structural networks in each group of Val homozygotes (blue) and Met-allele carriers (dark gray) and **(B)** three-dimensional representations (axial and sagittal views) of white matter network modules of each group. Each node is color-coded by the modular structures. The size of the nodes in **(A,B)** is in proportion to the number of degrees of each node. The nodes and edges of white matter structural networks were visualized using the BrainNet Viewer (Xia et al., 2013). Abbreviations: Val, valine; Met, methionine.

Modular organization of networks was also examined in group-averaged white matter structural networks of each group of Val homozygotes and Met-allele carriers and is presented in Figure 1. The group-averaged white matter structural network was reconstructed in each genotype group with a threshold to only include connections found in at least 50% of the subjects. The cell values of individual matrices were then averaged and converted to an unweighted matrix in a binary fashion. Both genotype groups showed a similar modular configuration, where bilateral fronto-parieto-occipital modules (orange and blue circles in Figure 1, respectively) and bilateral limbic modules (yellow and green circles in Figure 1, respectively) were identified in both Val homozygotes and Met-allele carriers.

A similar modular configuration was observed in both genotype groups. Bilateral fronto-parieto-occiptial modules (orange and blue circles in Figure 1, respectively) and bilateral limbic modules (yellow and green circles in Figure 1, respectively) were identified in both Val/Val and Met-allele carrier groups.

### Between-Group Differences in Network Robustness Measures

**Figure 2:**
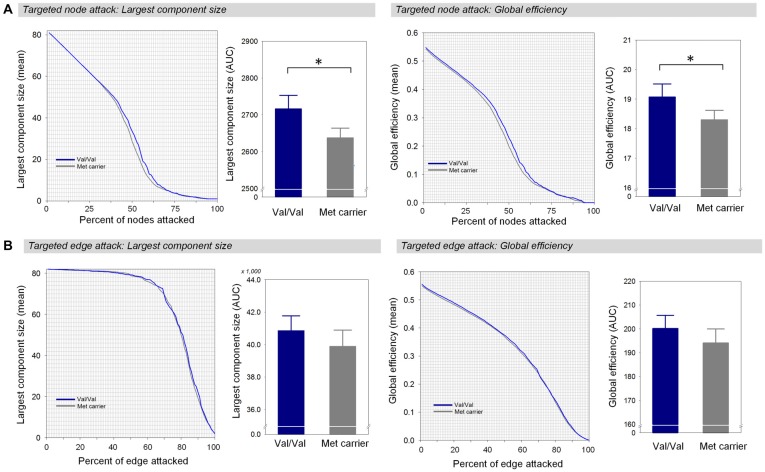
Network robustness of the white matter structural network in each group of Val homozygotes (blue) and Met-allele carriers (dark gray) in response to targeted node attacks **(A)** and targeted edge attacks **(B)**. The line graphs indicate changes in the largest connected component size (left panel) and global efficiency (right panel) as a function of nodes or edges removed according to their betweenness centrality in a decreasing order. The bar graphs show the comparisons of AUCs of the largest component size (left panel) or global efficiency (right panel) between Val homozygotes and Met-allele carriers. Asterisks indicate a significant group difference at Bonferroni-corrected *p* < 0.05. The error bars represent 95% confidence intervals. Abbreviations: Val, valine; Met, methionine; AUC, area under the curve.

When targeted attacks occurred on nodes or edges based on their betweenness centrality, the largest component size and global efficiency generally decreased (Figure 2). For targeted removal of nodes, the AUC of the largest component size was significantly greater in the Val homozygote group than in the Met-allele carrier group, even after the Bonferroni correction (*β* = 0.35, Bonferroni-corrected *p* < 0.05, effect size = 0.80, Figure 2A). Greater AUC of global efficiency was also found in the Val homozygote group in comparison to the Met-allele carrier group (*β* = 0.31, Bonferroni-corrected *p* < 0.05, effect size = 0.66, Figure 2A). These results suggest that the network vulnerability to targeted node attack was greater in the Met-allele carrier group in comparison to the Val homozygote group. There were no between-group differences in the AUCs of the largest component size (*β* = 0.15, Bonferroni-corrected *p* > 0.1, effect size = 0.29, Figure 2B) and global efficiency (*β* = 0.15, Bonferroni-corrected *p* > 0.1, effect size = 0.31, Figure 2B) in relation to the targeted edge attacks.

**Figure 3:**
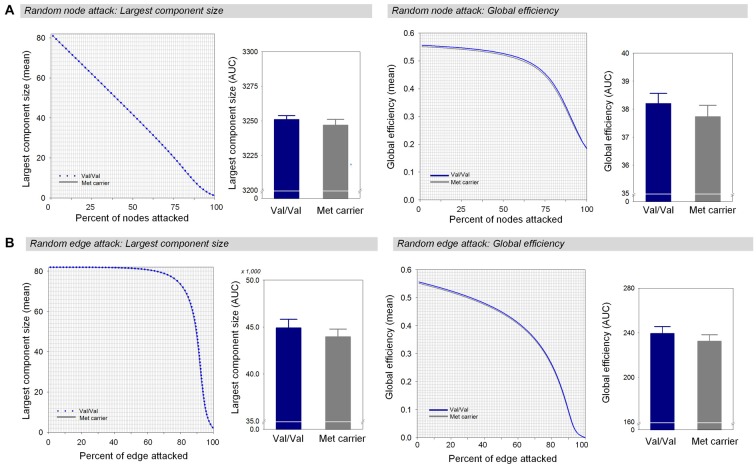
Network robustness of the white matter structural network in each group of Val homozygotes (blue) and Met-allele carriers (dark gray) in response to random node attacks **(A)** and random edge attacks **(B)**. The line graphs indicate changes in the largest connected component size (left panel) and global efficiency (right panel) as a function of randomly removed nodes or edges with 1000 permutations. The bar graphs shows the comparisons of AUCs of the largest connected component size (left panel) or global efficiency (right panel) between Val homozygotes and Met-allele carriers. The error bars represent 95% confidence intervals. Abbreviations: Val, valine; Met, methionine; AUC, area under the curve.

Under random failures, the largest component size and global efficiency generally decreased during continuous removal of nodes or edges (Figure 3). The AUCs of the robustness measures at the random node attack including the largest component size (*β* = 0.16, Bonferroni-corrected *p* > 0.1, effect size = 0.29, Figure 3A) and global efficiency (*β* = 0.17, Bonferroni-corrected *p* > 0.1, effect size = 0.34, Figure 3A) did not differ between the two genotype groups. In addition, there were no differences in the AUCs of the largest component size (*β* = 0.17, Bonferroni-corrected *p* > 0.1, effect size = 0.34, Figure 3B) and global efficiency (*β* = 0.17, Bonferroni-corrected *p* > 0.1, effect size = 0.34, Figure 3B) during random removal of edges.

Auxiliary analyses were performed to investigate between-group differences in the AUCs of global efficiency during targeted and random edge attacks to the weighted white matter structural networks. There were no differences in the AUC of robustness measure between the two genotype groups during the targeted edge attacks (*β* = 0.13, *p* = 0.27, effect size = 0.28, Supplementary Figure S1A) as well as the random edge attacks (*β* = 0.11, *p* = 0.36, effect size = 0.22, Supplementary Figure S1B).

In addition, we identified the nodes that had the greatest impact on the white matter structural network when they were removed, for both genotype groups (Supplementary Figure S2). Of these nodes, we found the 10 most influential nodes for both Val homozygotes and Met-allele carriers, and a similar configuration was observed for both groups.

We also repeated analyses using different thresholds of fiber number ranged from 2 to 4. These repeated analyses have demonstrated that the main results are not influenced by the effects of fiber number thresholds (Supplementary Table S1).

## Discussion

In the current study, we were interested in the effect of the BDNF Val66Met polymorphism on the configuration of the white matter structural network. Thus, the global network topology of the individual white matter structural networks in the undamaged state as well as the network robustness measures in the damaged state were compared between the Val homozygote and Met-allele carrier groups, respectively. There was no between-group difference in the global network measures including global efficiency, local efficiency, and modularity. However, we found the BDNF Val66Met polymorphism to have effects on the robustness of the white matter structural network. While the white matter structural network was comparably resilient to random failures between the two genotype groups, the Val homozygote group showed greater robustness of the white matter structural network under targeted attacks on central nodes.

Graph-theoretical analysis can successfully identify the differences in network configuration between healthy individuals and in patients with various brain diseases (Bassett and Bullmore, 2009). Graph-theoretical analysis has been also applied to reveal differences in the configuration of the brain network according to genetic variants as can be found in the apolipoprotein E (APOE) gene (Brown et al., 2011; Zhao et al., 2012). Besides the APOE gene, the BDNF gene is another common gene that exhibits the impact of genetic mutation on aspects of neuroplasticity.

The transition from Val to Met at codon 66 of the BDNF gene leads to decreased concentration of active BDNF protein products. Given that the BDNF plays an important role in the synaptic transmission involving glutamate N-methyl-D-aspartate receptors (Lu and Figurov, 1997; Woo et al., 2005), the BDNF Val66Met polymorphism has been suggested to modulate neural plasticity (Egan et al., 2003; Kleim et al., 2006; Cheeran et al., 2008). Likewise, changes in neural plasticity in terms of altered long-term potentiation or long-term depression have been observed in relation to the BDNF Val66Met polymorphism in the human brain (Cheeran et al., 2008; Antal et al., 2010) as well as in animal models (Lu and Figurov, 1997; Woo et al., 2005).

However, the influence of different genotypes of the BDNF gene on the network configuration of the human brain has remained unclear. The current study first sought to examine the effects of the BDNF Val66Met polymorphism on the topology of the white matter structural network by comparing global network measures as summaries of brain configuration. However, there were no topological differences that was observed in the undamaged state between Met-allele carriers and Val homozygotes. Additionally, considering the possible role of the the BDNF Val66Met polymorphism in manifesting neuroplasticity (Pascual-Leone et al., 2011), we examined whether the impact of the BDNF Val66Met polymorphism on network robustness could be revealed with respect to the distinguished potential for neuroplasticity. Our results showed that the two genotype groups had significant differences in network robustness under targeted attacks, but not under random failures.

Error tolerance represented robustness to random failures simulated by randomly eliminating nodes and edges that applied to gray matter regions and interregional white matter connections, respectively. The two genotype groups had equally resilient white matter structural networks under random failures. In both genotype groups, the shape of the robustness curve depended on the measures chosen to evaluate network robustness. For instance, global efficiency remained above 90% of the value up until 60% of the nodes were removed, whereas the largest component size linearly decreased as low as 40% of the value for the undamaged network.

Attack tolerance addressed robustness to targeted removal of pivotal nodes or edges that could be detected based on different criteria. In the current study, we considered the central structure of the white matter structural network to choose important nodes and edges. Compared to random failures, global efficiency decreased more rapidly in response to damage to nodes with high betweenness centrality. In particular, global efficiency decreased more drastically in the Met-allele carrier group, leading to a significant difference in the AUC between the two genotype groups. As previous research indicated that the brain functional network reveals greater vulnerability to targeted attacks than random failures (Achard et al., 2006), the current findings indicate that such a phenomenon could be replicated for the white matter structural network. Additionally, between-group differences in network robustness against targeted damage to nodes were observed in terms of the largest component size.

Although there appears to be some inconsistency in the findings (Kohannim et al., 2012; Hayashi et al., 2014), previous imaging genetics studies using DTI methods have suggested effects of the BDNF Val66Met polymorphism on white matter structures (Chiang et al., 2011; Tost et al., 2013; Forde et al., 2014). For instance, Met-allele carriers showed greater fractional anisotropy values (Chiang et al., 2011) and lower radial diffusivity values (Tost et al., 2013) in the corpus callosum, prefrontal and occipital areas, as compared with Val homozygotes. These findings suggest that there is a lesser impact on white matter structures in Met-allele carriers relative to Val homozygotes. Likewise, we found that the white matter structural network of Met-allele carriers may be approximately robust to targeted edge attacks as that of Val homozygotes. It may be emphasized that Met-allele carriers are not always inferior to Val homozygotes in network resilience to targeted attacks. Indeed, the Met allele appears to be favorable in some cases of neurodevelopment and neurodegeneration (Montag et al., 2009), such that it can even exhibit protective effects lacking in the Val allele (Pezawas et al., 2008).

In sum, the white matter structural network was equally robust to random failures in both genotype groups, but the white matter structural network was more vulnerable to targeted attacks on central nodes in Met-allele carriers. It is noteworthy, that simulations of damage or lesions in gray matter regions or white matter connections considered in this study may not be exactly reflect real situations. However, our findings provide possible connections to previous observations regarding the impact of the BDNF Val66Met polymorphisms on gray and white matter structures, and may enable us to generate a hypothesis about the variations of the potential for neuroplasticity according to the genotypes of the BDNF gene. Depending on whether damage occurs in gray matter regions or white matter connections, and whether damaged gray matter regions or white matter connections are pivotal or peripheral, the effect of the BDNF Val66Met polymorphism on the robustness of the white matter structural network may be distinguishable. This then may provide insight regarding the brain’s susceptibility to brain disorders. For instance, the fact that Met-allele carriers generally have higher susceptibility to brain disorders (Sklar et al., 2002; Neves-Pereira et al., 2005; Verhagen et al., 2010) could be based on the inherent organization of their white matter structural network that exhibits greater vulnerability to damage in the pivotal gray matter regions, along with a higher likelihood of structural impact on the gray matter regions.

However, when considering possible interactions between the BDNF Val66Met polymorphism and other genetic variants (Pezawas et al., 2008), we are aware that the influence of multiple genetic variants on brain configuration needs to be further explored. Furthermore, it should be delineated whether genetic variant-dependent differences in brain configuration may be observed since birth or it could be settled through the continuous modulation of experience-dependent neuroplasticity (Kleim et al., 2006) during development, although either of these may be regarded as being genetically inherent.

Several limitations should be noted in the current study. First of all, the number of participants in this study is relatively small compared to other neuroimaging genetics studies. However, it is noteworthy that there were statistically significant group differences in network robustness revealed even after employing a Bonferroni-correction for multiple comparisons. In addition, different approaches in graph-theoretical analysis could not be thoroughly considered for the validation of our findings. Although preliminary findings regarding the effects of the BDNF Val66Met polymorphism on the robustness of the weighted white matter structural network have been provided as an auxiliary analysis, it should be noted that the white matter structural network with unweighted edges was our primary interest in order to investigate the network endophenotype of the BDNF Val66Met polymorphism. Future studies using the white matter structural network with edges weighted by fiber tract count or white matter integrity (van den Heuvel and Sporns, 2011) are warranted. Similar to other studies examining white matter structural network using deterministic tractography, the well-known issue of crossing fiber should be considered in interpreting the findings (Mori and van Zijl, 2002). As for the robustness measures, we employed popular measures that have been used in previous studies on network resilience. However, there are definitely other measures of robustness, such as communicability (Estrada and Hatano, 2008) that was suggested to be a promising measure for assessing the effect of simulated attacks in the brain network (de Reus and van den Heuvel, 2014), and may be further tested in the future. It should also be noted that the ancestry information, whether by using single nucleotide polymorphism or mitochondrial haplotypes, was not assessed in the current study. However, all participants reported their ancestry as Korean. Future studies considering genetic ancestry information would be necessary to investigate the network-based brain endophenotypes of the BDNF Val66Met polymorphism.

## Author Contributions

The following persons have contributed to the present manuscript as authors according to the journal’s guidelines for authorship: CP, JK, EN, D-WL, GHK, MK, NK, TDK, SK, IKL and SY. CP and JK contributed equally to the manuscript.

## Conflict of Interest Statement

The authors declare that the research was conducted in the absence of any commercial or financial relationships that could be construed as a potential conflict of interest.

## Footnotes

^1^http://trackvis.org/

^2^http://surfer.nmr.mgh.harvard.edu

^3^http://www.brain-connectivity-toolbox.net
