# Supplementary material for: The BDNF Val66Met Polymorphism Affects the Vulnerability of the Brain Structural Network
Source: Front Hum Neurosci. 2017 Aug 3;11:400. doi: 10.3389/fnhum.2017.00400 (PMC5541016; doi:10.3389/fnhum.2017.00400)
Supplement: Supplementary file 1 [file Table_1.pdf]

Supplementary Table. Group difference in network measures using different fiber number thresholds

Network measures	β (p values)		
	Fiber number threshold = 2	Fiber number threshold = 3	Fiber number threshold = 4
Between-group differences in global network measures			
Global efficiency	0.18 (0.12)	0.17 (0.15)	0.16 (0.16)
Local efficiency	-0.02 (0.88)	-0.05 (0.70)	-0.09 (0.44)
Modularity	-0.23 (0.05)	-0.20 (0.10)	-0.21 (0.08)
Between-group differences in network robustness measures			
Targeted node removal			
Global efficiency	0.29 (0.01)	0.31 (0.008)	0.30 (0.009)
Largest component size	0.29 (0.01)	0.35 (0.003)	0.38 (0.001)
Targeted edge removal			
Global efficiency	0.17 (0.14)	0.15 (0.19)	0.15 (0.20)
Largest component size	0.18 (0.13)	0.15 (0.21)	0.13 (0.26)
Random node removal			
Global efficiency	0.19 (0.11)	0.17 (0.15)	0.17 (0.15)
Largest component size	0.16 (0.18)	0.16 (0.18)	0.15 (0.18)
Random edge removal			
Global efficiency	0.19 (0.11)	0.17 (0.14)	0.17 (0.16)
Largest component size	0.18 (0.11)	0.17 (0.14)	0.17 (0.16)

For the assessment of group differences in global network measures (global efficiency, local efficiency, and modularity), multiple linear regression analysis was used after adjusting for age. Group differences in network robustness measures (areas under the curve of the largest component size and global efficiency at node and edge attacks) were examined using the multiple regression analysis after adjusting for age.
