# Supplementary material for: The BDNF Val66Met Polymorphism Affects the Vulnerability of the Brain Structural Network
Source: Front Hum Neurosci. 2017 Aug 3;11:400. doi: 10.3389/fnhum.2017.00400 (PMC5541016; doi:10.3389/fnhum.2017.00400)
Supplement: Supplementary file 2 [file Image_1.pdf]

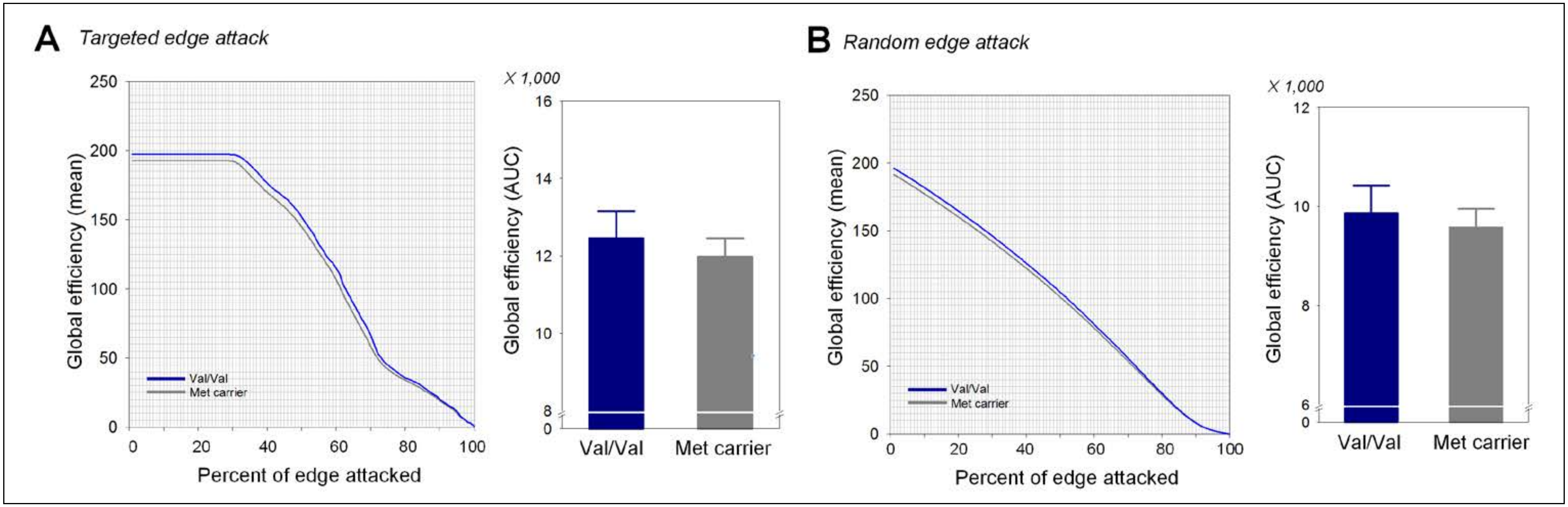

Supplementary Figure 1. Targeted edge attacks (A) and random edge attacks (B) to the weighted white matter structural network in each group of Val homozygotes (blue) and Met-allele carriers (dark gray). The line graphs indicate the changes in global efficiency as a function of targeted (A) and random (B) removal of edges. The bar graphs show the comparisons of AUCs of global efficiency between Val homozygotes and Met-allele carriers. The error bars represent 95% confidence intervals.

Abbreviations: Val, valine; Met, methionine; AUC, area under the curve.
