# Supplementary material for: The BDNF Val66Met Polymorphism Affects the Vulnerability of the Brain Structural Network
Source: Front Hum Neurosci. 2017 Aug 3;11:400. doi: 10.3389/fnhum.2017.00400 (PMC5541016; doi:10.3389/fnhum.2017.00400)
Supplement: Supplementary file 3 [file Image_2.pdf]

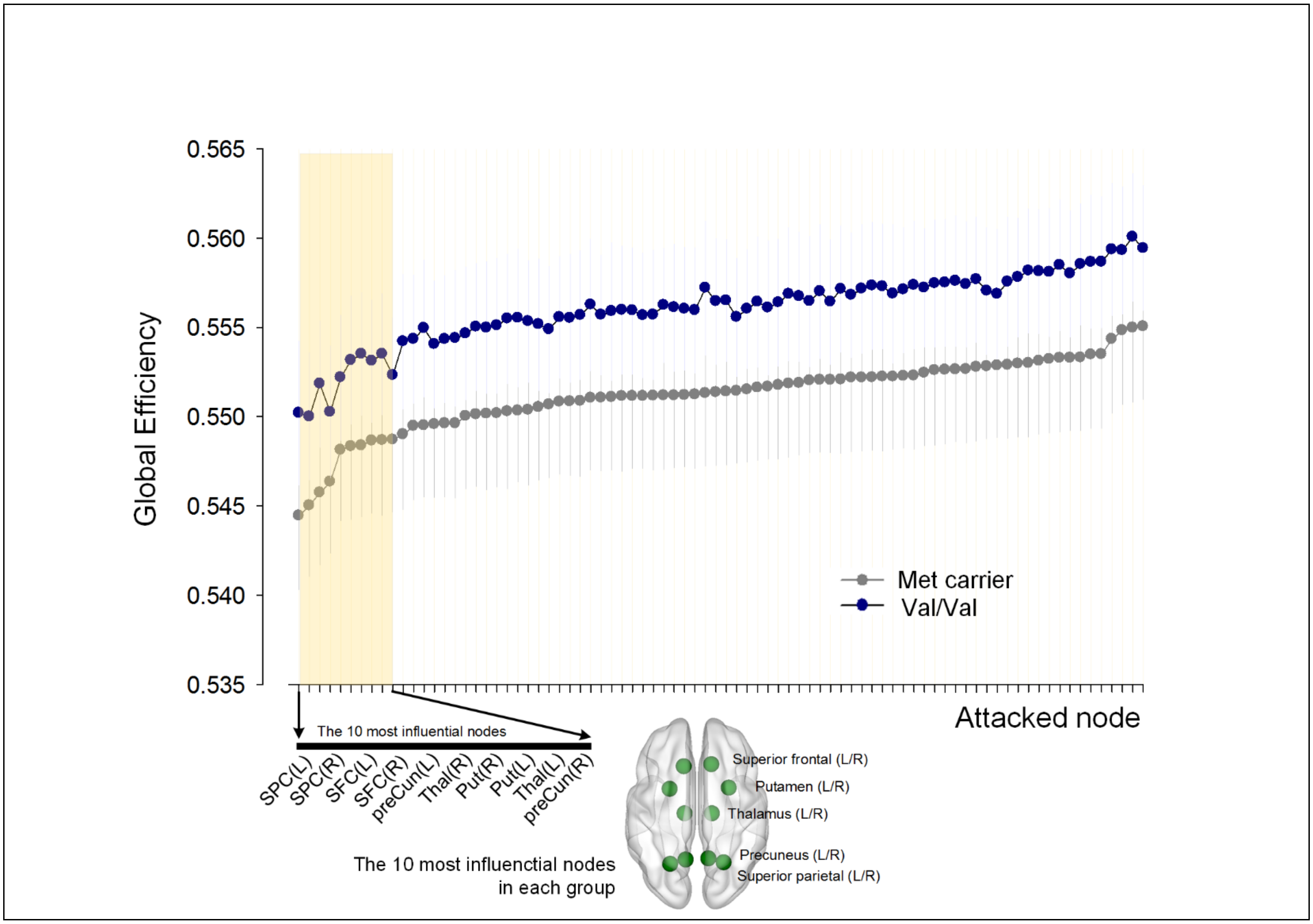

Supplementary Figure 2. The mean values of global efficiency at the attack of each node are displayed as blue (Val homozygotes) and dark gray (Met-allele carriers) circles in increasing order. Three-dimensional rendering of the 10 most influential nodes for both genotype groups, which have the biggest impact on the network against their removals, is represented as green circles in the standard space brain using the BrainNet viewer (Xia et al. 2013).

Abbreviations: Val, valine; Met, methionine; SPC, superior parietal cortex; SFC, superior frontal cortex; preCun, precuneus; Thal, thalamus; Put, putamen; L, left; R, right.
